# Energy-splitting from persistent luminescence nanoparticles with trivalent Cr ions for ratiometric temperature sensing†

**DOI:** 10.1039/d4ra04618h

**Published:** 2024-08-29

**Authors:** Tian-Qi Zhao, Renagul Abdurahman, Xue-Bo Yin

**Affiliations:** a Xinjiang Key Laboratory of Novel Functional Materials Chemistry, College of Chemistry and Environmental Science, Kashi University Kashi Xinjiang 844000 China renagul111@aliyun.com; b Institute for Frontier Medical Technology, College of Chemistry and Chemical Engineering, Shanghai University of Engineering Science Shanghai 201620 China xbyin@nankai.edu.cn xbyin@sues.edu.cn

## Abstract

MgGa_2_O_4_ (MGO) with the spinel structure exhibits abundance defects and could achieve the modulation of emission by ion doping as persistent luminescence nanoparticles (PLNPs). Here, we introduced Cr^3+^ ions into MGO to achieve near-infrared (NIR) emission, and Pr^3+^ ions to tune the lattice environment for enhanced NIR emission. The optimal composite, MgGa_2_O_4_: 0.005Cr^3+^, 0.003Pr^3+^ (MGCP), achieved enhanced NIR emission at 709 nm under 222 nm excitation. The concentration quenching was observed due to electric dipole–quadrupole interaction at high Cr^3+^ and Pr^3+^ content. The afterglow mechanism was revealed, while the energy-splitting occurs from trivalent Cr^3+^ ions at 650 and 709 nm, thanks to the complex lattice environment. We observed that the emission at 709 nm decreased, while the satellite signal at 650 nm increased first and then decreased intensity with increasing temperature, due to the intervalence charge transfer for Cr^3+^ ions at 303–528 K. Ratiometric temperature sensing was therefore realized with superb linearity, high absolute sensitivity at 303 K for 4.18%, and accuracy at 528 K for 2.62 K, confirming with the luminescence intensity ratio at 709 and 650 nm under excitation at 222 nm. Thus, we provide a method with energy-splitting emission of Cr^3+^ ions to design temperature sensing.

## Abstract 2

MgGa_2_O_4_ (MGO) with positive and negative spinel structure exhibits abundance defects, achieving the modulation of emission by ion doping as persistent luminescence nanoparticles (PLNPs).

## Graphical abstract

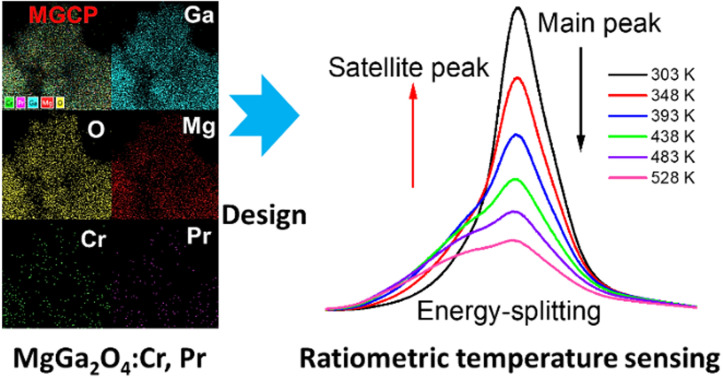

## 1. Introduction

Temperature, as an important physical parameter, has been widely used in science and research.^1^ Contact temperature sensing shows slow response with limited applications.^2^ Thus, various non-contact sensing approaches have been developed, such as that with metal–organic frameworks,^3^ upconverted phosphors,^4^ and carbon dots as temperature probes.^5^ Dual-emission could achieve luminescence intensity ratio (LIR) for ratiometric temperature sensing applications with improved precision,^6,7^ but the design of dual-emission materials is critically required for LIR temperature sensing.

Persistent luminescence nanoparticles (PLNPs) are composed of hosts, emission centers, and traps, while the stored energy is gradually release as afterglow emission.^8,9^ The afterglow emission could achieve interference-free sensing and imaging, while information storage, encryption, anti-counterfeiting, and even temperature sensing have been reported.^10–12^ The host achieves the modulated optical performance by an ion-doping strategy.^13,14^ Bi^3+^ ions, as emission centers, were doped into MgGa_2_O_4_ (MGO) to achieve the emission at 410 nm from octahedral Bi^3+^, 500 nm from MGO intrinsic defects, and 709 nm from tetrahedral Bi^3+^ for LIR temperature sensing.^15^ The previously reported energy levels ^2^E → ^4^A_2_ at 710 nm and ^4^T_2_ → ^4^A_2_ at 800 nm of Cr^3+^ ions were designed for LIR temperature sensing.^16^ Further, Cr^3+^ and Zn^2+^ ions were doped into the MGO to get unique optical behavior, as the excited state energy level ^2^E from Ga^3+^ ions showed energy-splitting as ^2^E_A_ → ^4^A_2_ at 505 nm and ^2^E_B_ → ^4^A_2_ at 425 nm.^17^ The rare earth ions with abundant energy levels have been verified to enhance the emission.^18,19^ Thus, energy-splitting emission could be developed for ratiometric temperature sensing, especially using Cr^3+^ ions doped MGO systems, while the mechanism is also interesting for energy-splitting.

Herein, we introduced Cr^3+^ ions into MgGa_2_O_4_ to achieve NIR emission at 709 nm under 222 nm excitation and Pr^3+^ ions to enhance the emission with the optimal composite, as MgGa_2_O_4_: 0.005Cr, 0.003Pr (MGCP). The effect of Cr^3+^ and Pr^3+^ ions on the optical behaviour was investigated, while concentration quenching was used to explain the optimal composite at high Cr^3+^ and Pr^3+^ content. The afterglow mechanism revealed that energy-splitting occurred from Cr^3+^ ions, while Pr^3+^ ions changed the lattice environment to enhance the NIR emission. The study on the response mechanism revealed that the emissions at 709 nm and the satellite peak were derived from the energy-splitting for Cr^3+^ ions. Upon temperature rises, we observed the decreased intensity for the emission at 709 nm, while the satellite peak at 650 nm increased first and then decreased gradually. Due to the intervalence charge transfer that occurred among the Cr^3+^ ions, the decreased intensity for the emission at 709 nm and the increased satellite signal occur, simultaneously. Thus, ratiometric temperature sensing was proposed with the two emissions at 709 and 650 nm for MGCP in the temperature range 303–528 K. Thus, we reported that the energy-splitting emissions of Cr^3+^ ions were used for ratiometric temperature sensing application.

## 2. Experimental section

### 2.1 Materials and synthesis

MgCl_2_·6H_2_O was purchased from Tianjin Windship Chemical Reagent Technology Company Limited. Ga_2_O_3_ (99.99%), Cr(NO_3_)_3_·9H_2_O (99.99%), and Pr(NO_3_)_3_·6H_2_O (99.99%) were obtained from Shanghai Aladdin. HNO_3_ (68%) and ammonia (28%) were obtained from Tianjin Guangfu Fine Chemicals Research Institute. Pure water was acquired from Hangzhou Wahaha Company.

MgCl_2_·6H_2_O, Cr(NO_3_)_3_·9H_2_O, and Pr(NO_3_)_3_·6H_2_O were dissolved in pure water to obtain 0.2 mol L^−1^ Mg^2+^, 0.01 mol L^−1^ Cr^3+^, and 0.01 mol L^−1^ Pr^3+^, respectively. The 0.2 mol L^−1^ Ga^3+^ solution was prepared based on the previous report.^20^ Mg^2+^, Ga^3+^, Cr^3+^, and Pr^3+^ solutions were sequentially added to a round-bottom flask and stirred for 0.5 h as uniform solution. The pH was adjusted to 9 with 28% ammonia and stirred for 2 h. The products were then washed with pure water and dried under vacuum at 100 °C for 3 h. After calcined at 800 °C for 3 h, MgGa_2_O_4_: *x*Cr^3+^*y*Pr^3+^ (*x* = 0, 0.001, 0.003, 0.005, 0.007, 0.009; *y* = 0, 0.001, 0.003, 0.005, 0.007, 0.009) PLNPs were obtained. The PLNPs with optimal composition were taken for ratiometric temperature sensing application.

### 2.2 Characterization

The Bruker D8 Focus Advance X-ray diffractometer (Bruker, Germany) with CuKα radiation recorded X-ray diffraction (XRD) pattern. The JEM 2100 F (JEOL, Japan) with X-MAS (Oxford, England) was used to get energy dispersive spectrometer (EDS) and transmission electron microscope (TEM) patterns. The BeNano 90 (Better, China) was used to obtain hydrated particle size distribution pattern. Thermo SCIENTIFIC ESCALAB 250Xi (Thermo, America) recorded X-ray photoelectron spectroscopy (XPS) pattern. F-7100 fluorescence spectrophotometer (HITACHI, Japan) was used to obtain photoluminescence spectra and variable temperature emission spectroscopy pattern. FS5C fluorescence spectrophotometer (Edinburgh, England) was used to record the afterglow decay curves pattern. The Bruker EMXPlus (Bruker, Germany) recorded electron paramagnetic resonance (EPR) pattern. TOSL-3DS (Guangzhou Rongfa, China) recorded thermoluminescence pattern. UV-visible/NIR Spectrophotometer UH4150 (HITACHI, Japan) was used to obtain diffuse reflectance spectra (DRS) pattern.

## 3. Results and discussion

### 3.1 Structure characterization

**Fig. 1:**
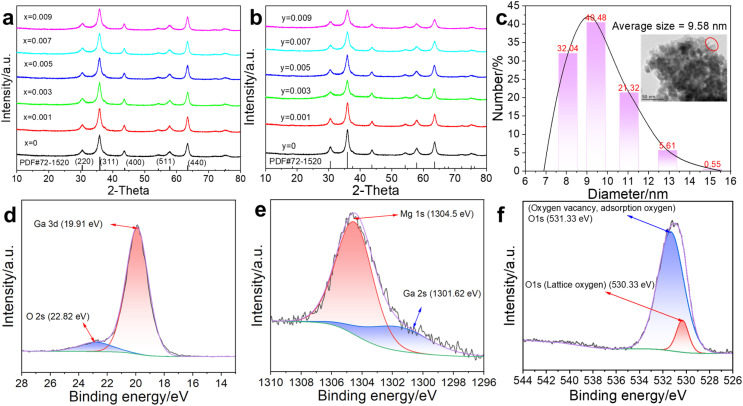
XRD patterns of (a) MGO: *x*Cr^3+^ and (b) MGO: 0.005 Cr^3+^, *y*Pr^3+^. (c) Number particle size distribution (the inset is TEM with 50 nm scale) of MGCP. High-resolution XPS spectrum patterns in MGCP of (d) Ga 3d, (e) Mg 1s, (f) O 1s.

MGCP were characterized to reveal the effect of Cr^3+^ and Pr^3+^ ions. As shown in Fig. 1a and b, the XRD patterns of MGO: *x*Cr^3+^, *y*Pr^3+^ exhibited the diffraction peaks at 30.52°, 35.97°, 43.69°, 54.23°, 57.81° and 63.53° for crystal planes (220), (311), (400), (422), (511), and (440), similar to that from the standard card PDF#72-1520 for MGO.

TEM images reveal the information, such as the size, crystal orientation, lattice defects, and chemical composition.^20^ The TEM showed that the average size of MGCP was 9.58 nm (Fig. 1c). The high-resolution TEM image provided the lattice fringes, such as the crystal faces (311) and (400), for the lattice spacings of 2.56 and 2.14 Å (Fig. S1a†). Moreover, Cr^3+^ and Pr^3+^ ions were evenly distributed in MGCP as observed from the EDS element mapping pattern (Fig. S1b†). Thus, MGCP with spinel structure was successfully synthesised for further experiment.

High-resolution XPS spectra were used to reveal the valence states of metal ions in MGCP. The C element was introduced into MGCP and observed with the peaks at 284.8, 286.07, and 288.75 eV for C–C (C 1s), C–O–C, and O–C=O (Fig. S1c†).^21^ The signal at 284.8 eV was used as position correction for the other elements, as Ga, Mg, and O. Ga 3d exists at 19.91 eV for trivalent Ga (Fig. 1d).^22^ Mg 1s was observed at 1304.5 eV as the divalent Mg (Fig. 1e).^23^ O 1s was observed at 530.33 and 531.33 eV as the lattice oxygen and oxygen vacancy (Fig. 1f).^22^ Thus, Cr^3+^ and Pr^3+^ ions did not affect the structure and valence states of MGO due to their low content.

### 3.2 Optical response

**Fig. 2:**
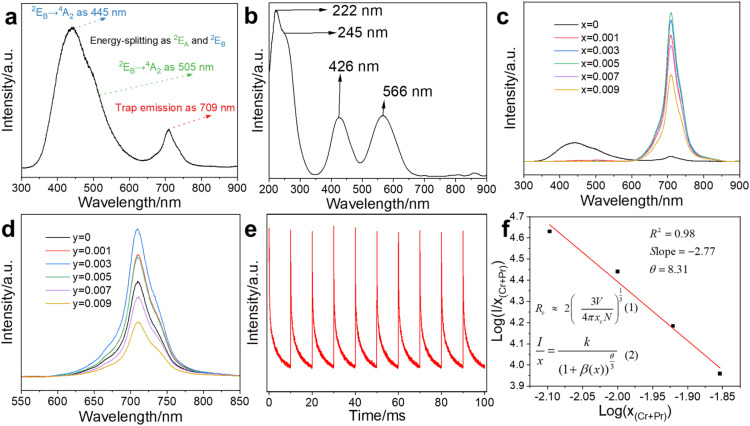
(a) Emission spectrum of MGO at 222 nm excitation. (b) Excitation spectrum of MGCP for the emission at 709 nm. Emission spectrum of (c) MGO: *x*Cr^3+^ and (d) MGO: 0.005 Cr^3+^, *y*Pr^3+^ at 222 nm excitation. (e) Afterglow lifetime at the room temperature within 100 ms using a 100 Hz microsecond Flashlamp patterns of MGCP. (f) The linear relationship between log(*x*_(Cr+Pr)_) and log(*I*/*x*_(Cr+Pr)_) to verify concentration quenching.

MgGa_2_O_4_ (MGO) exhibits abundance intrinsic defects and could modulate its luminescence properties by the introduction of doped ions.^10,13^ MGO showed the emission at 445 nm with broadband around 350–600 nm under 222 nm excitation (Fig. 2a), from deformed octahedron coordination of Ga^3+^ ions as ^2^E_A_ → ^4^A_2_ at 505 nm and ^2^E_B_ → ^4^A_2_ at 445 nm,^15^ while the 709 nm emission was attributed to the oxygen vacancy.^17^ Thus, we introduced Cr^3+^ ions into MGO to achieve NIR emission, while Pr^3+^ ions enhance NIR emission with the optimal composite for MGCP. The excitation was observed at 222, 245, 426, and 566 nm for MGCP (Fig. 2b). The excitation at 222 nm was originated transition from Ga–O, while that at 245, 426, and 566 nm from was attributed to the ^4^A_2_(^4^F) → ^4^T_1_(^4^P), ^4^A_2_(^4^F) → ^4^T_1_(^4^F), and ^4^A_2_(^4^F) → ^4^T_2_(^4^F) spin-allowed transitions of Cr^3+^ ions, respectively.^24^ MGCP exhibited the NIR emission as the electron transfer of ^2^E → ^4^A_2_(^4^F) of Cr^3+^ ions.^25^ The strong excitation at 222 nm was validated with the absorption of MGCP and selected to explore the optical behaviour and temperature sensing application.

The emission at 709 nm with the broadband from 600 to 850 nm and afterglow lifetime of MGO: *x*Cr^3+^ first increased and then decreased with the optimal content (Fig. 2c, S2a and b†), *x* = 0.005, as MGO: 0.005Cr^3+^ (MGC). Thus, MGC was selected to enhance the emission by the introduction of Pr^3+^ ions. As shown in Fig. 2d, the emission intensity of MGO: 0.005Cr^3+^, *y*Pr^3+^ first increased and then decreased as Pr^3+^ content increased with the optimal content (Fig. S2c†), *y* = 0.003, as MGO: 0.005Cr^3+^, 0.003Pr^3+^ (MGCP). Cr^3+^ and Pr^3+^ replace Ga^3+^ with the octahedron coordination mode due to their same valence state.^26^ Pr^3+^ ions, as rare earth element with abundant energy levels, changed crystal field environment around Cr^3+^ ions and thus enhanced the emission at 709 nm.^8^ The afterglow of MGCP could be repeated excitation within 100 ms for 10 times at room temperature (Fig. 2e), so MGCP has excellent photo-stability and could be repeated for temperature sensing.

### 3.3 Concentration quenching

Cr^3+^ and Pr^3+^ with high content could quench the emission of MGCP as the concentration quenching mechanism.^15^ The critical distance (*R*_c_) between the emission centers is a key parameter for concentration quenching effect and can be calculated with eqn (1) in Fig. 2f,^27^ where *V* is the unit cell volume of MGO, *X*_c_ is the optimal concentration of MGCP as 0.008, and *N* is Cr^3+^ and Pr^3+^ replacement number to Ga^3+^ lattice sites in unit cell. Specifically, for *V* = 567.3 Å^3^, *X*_c_ = 0.008, and *N* = 16, *R*_c_ is calculated as 20.38 Å.

Exchange and multipolar interactions are used to explain the concentration quenching effect. Typically, *R*_c_ is limited to 5–8 Å for the exchange interaction mechanism.^28^ Obviously, the actual *R*_c_ of 20.38 Å is much larger than 5–8 Å. Thus, multipolar interaction is dominant for the concentration quenching. The Dexter theory can evaluate the type of multipolar interaction with eqn (2) in Fig. 2f,^29^ where *x* is the content of Cr^3+^ and Pr^3+^ ions, *I* is the emission intensity, *k* and *β* are the constants for specific interaction in a given host. The *θ* values are 6, 8, and 10, for electric dipole–dipole (d–d), dipole–quadrupole (d–q), and quadrupole–quadrupole (q–q) interactions, respectively. After the linear fit curve of log(*I*/*x*) to log(*x*), *θ* is obtained and close to 8 (Fig. 2f), so the quenching mechanism is related to the electric d–q interaction for the concentration quenching at high Cr^3+^ and Pr^3+^ content.

### 3.4 Afterglow mechanism

To verify the trap types for the emission, electron paramagnetic resonance (EPR) spectrum was recorded for MGCP (Fig. S3a†). The value at *g* = 1.99 belongs to the oxygen vacancy,^30^ while that at *g* = 3.85 is from Cr^3+^ in octahedral site.^31^ Thermoluminescence with the peak at 371 K showed trap energy level for MGCP with the single trap center from oxygen vacancy (Fig. S3b†). The empirical equation *E* = *T*_m_/500 (*E* stands for the trap energy level and *T*_m_ is the temperature of the thermoluminescence peak^20^) was used to calculate the trap energy level as 0.742 eV.

The DRS for MGCP could calculate the energy band (*E*_g_) according to the equations in Fig. S3c.^17^† Where *hv* is the photon energy defined as *hv* = 1240/*λ*. *A* is a constant and *F*(*R*) is defined as *F*(*R*_∞_) = (1 − *R*)^2^/*R*. When [*F*(*R*_∞_)*hv*]^2^ = 0, the *E*_g_ value was 4.75 eV for MGCP, but the impurity energy band existed for 3.03 eV, causing by ions doped or misalignment defects in the host,^32^ suggesting that the MGCP has a suitable lattice environment for afterglow emission.

**Fig. 3:**
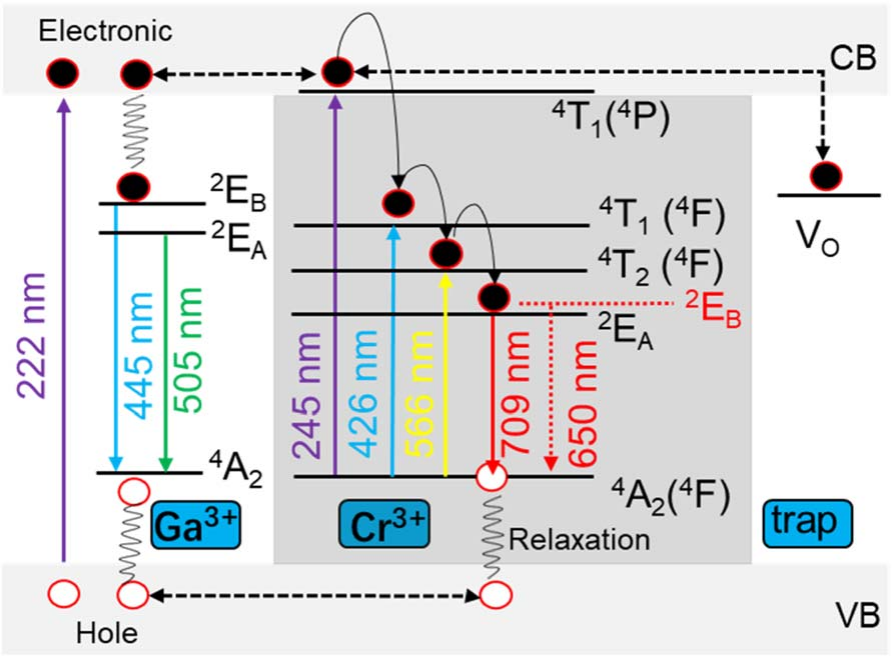
Schematic diagram of afterglow mechanism and energy-splitting of MGCP.

The excited electrons in valence band (VB) are trapped by the traps near the conduction band (CB) after the excitation at 222 nm, so the holes generate in the VB as shown in Fig. 3. The electrons are trapped and then slowly released to produce afterglow at 709 nm for Cr^3+^ ions with the combination of electrons and holes by relaxation effect. Moreover, energy transfer occurs from the MGO host to Cr^3+^ ions,^17,20^ as the emission at 445 nm disappear after the introduction of Cr^3+^ ions. Pr^3+^ ions increased effective traps by changing the crystal field environment around Cr^3+^ ions and thus enhanced the emission at 709 nm. The emission occurs for MGO from deformed octahedron coordination of Ga^3+^ ions as ^2^E_A_ → ^4^A_2_ at 505 nm and ^2^E_B_ → ^4^A_2_ at 445 nm.^15^ While energy level ^2^E → ^4^A_2_ from Cr^3+^ ions showed energy-splitting as ^2^E_A_ → ^4^A_2_ at 709 nm and ^2^E_B_ → ^4^A_2_ at 650 nm.

### 3.5 Ratiometric temperature sensing

**Fig. 4:**
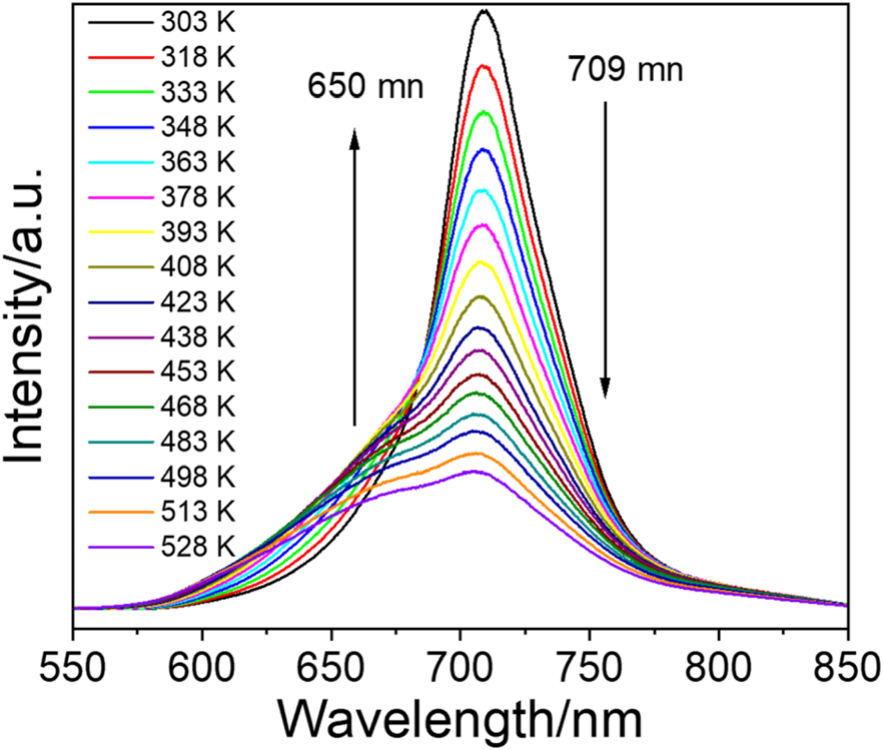
Emission spectra of MGCP under 222 nm excitation at the temperature from 303–528 K.

MGCP showed decreasing emission intensity at 709 nm with increasing temperature in the range of 303–528 K, while the intensity of satellite peak increased first and then decreased (Fig. 4 and S4a†).^18^ Due to the intervalence charge transfer occurred among the Cr^3+^ ions, the decreased intensity for the emission at 709 nm and the increased one for the satellite signal occur, simultaneously.^33^ The image profiles are plotted for MGCP that directly visualize the temperature change with the emissions (Fig. S4b†), so single emission center could be used for the development of ratiometric temperature sensing with the energy-splitting signal. Thus, we realized the LIR temperature sensing with the main emission at 709 nm and the satellite signal at 650 nm for MGCP as the temperature probe.

**Fig. 5:**
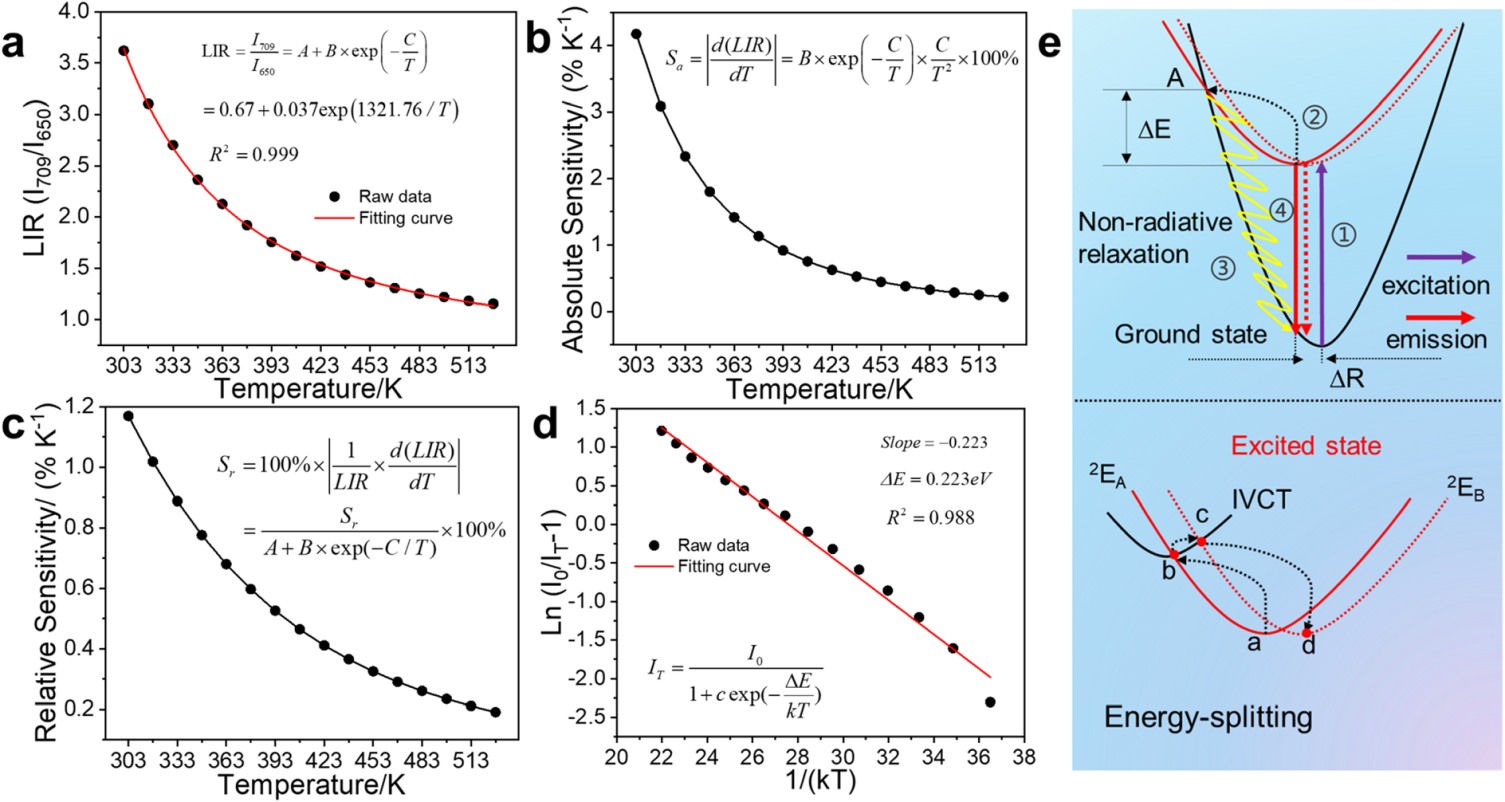
(a) LIR thermometry as a temperature linear relationship, (b) absolute and (c) relative sensitivity, and (d) the relationship of ln[(*I*_0_/*I*_*T*_) − 1] to 1/(*kT*) of MGCP under 222 nm excitation at the temperature from 303 to 528 K. (e) The thermal quenching of MGCP explained by configuration coordinate model and intervalence charge transfer.

The LIR performance of MGCP for temperature sensing was validated with the equation in Fig. 5a,^15^ Where *A*, Δ*E*, *k* are constants, and *T* is the absolute temperature. *I*_708_ and *I*_650_ are the emission intensity from MGCP under 222 nm excitation. The LIR with the ratio of *I*_708_ and *I*_650_ exhibits excellent temperature-dependent linear response with *R*^2^ as 0.999 (Fig. 5a).

The absolute sensitivity (*S*_a_) and relative sensitivity (*S*_r_) were measured to reveal the LIR temperature sensing performance.^19^*S*_a_ increased with the maximum at 303 K as 4.18%, while *S*_r_ decreased with the maximum at 303 K as 1.17%, according to the equations in Fig. 5b and c.^15^ The representative *S*_a_ and *S*_r_ of temperature sensing materials are illustrated in Table S1.† Our work exhibits similar and reliable performance than the previous results.

Temperature resolution (δ*T*) is another crucial parameter to assess the temperature sensing performance, according to equations in Fig. S4c,^7^† where the δ*Δ*/*Δ* is the typical value of 0.5% for the relative error in measurement.^34^ The δ*T* increased from 303 to 528 K and the maximum was observed at 528 K as 2.62 K and the minimum at 303 K as 0.43 K, so MGCP exhibits excellent temperature sensing accuracy. Ratiometric temperature sensing of MGCP was cycled between 303 and 528 K for 10 times. The result in Fig. S4d† illustrated that less deviation was observed, so MGCP exhibits high photo- and thermal-stability with excellent reproducibility for temperature sensing.

### 3.6 Thermal quenching for temperature response

The weakening of the emission at 709 nm with increasing temperature occurs due to the thermal quenching from the activation energy Δ*E* in non-radiative transition from the excited state to the ground state of Cr^3+^ ions. Δ*E* is calculated from the Arrhenius equation in Fig. 5d,^28^ where *I*_0_ and *I*_*T*_ are the emission intensity at the initial temperature and temperature *T*, respectively, while *c* is a constant, Δ*E* is the activation energy with this process, and *k* is the Boltzmann constant (8.629 × 10–5 eV K^−1^). A graph of ln[(*I*_0_/*I*_*T*_) − 1] as a function of 1/(*kT*) is plotted for MGCP. Δ*E* is calculated as 0.223 eV from the slope, so MGCP has low activation energy for temperature sensing for the fast response with high sensitivity.

The configuration coordinate model is further used to explain thermal quenching of MGCP for the emission at 709 nm from Cr^3+^ ions (Fig. 5e). The electrons were excited from ground state ^4^A_2_(^4^F) to excited state ^2^E (Process 1 in Fig. 5e).^8^ The first part of excited electrons reached the A-point by relaxation (Process 2 in Fig. 5e), leading to thermal quenching as the temperature increases by a non-radiative leap (Process 3 in Fig. 5e),^18^ while the second part of excited electrons returned from the excited state ^2^E to the ground state ^4^A_2_(^4^F) to achieve luminescence (Process 4 in Fig. 5e). The third part of the excited electrons transfer energy from ^2^E_A_ to ^2^E_B_ along the paths a–b–c–d in Fig. 5e, due to the intervalence charge transfer (IVCT) occurred among the Cr^3+^ ions.^35^ Thus, emission at 709 nm diminished along with temperature, while that at 650 nm suffers from IVCT as thermal excitation to be enhanced first and then weakened.^33^ Δ*R*, as the position difference of electrons before and after excitation in the relaxation process,^7^ realizes the broadband energy-splitting emission for Cr^3+^ ions. Thus, temperature sensing with energy-splitting emission was designed with excellent linearity, high sensitivity, and accuracy.

## 4. Conclusion

In conclusion, MGO with NIR emission at 709 nm was obtained by the introduction of Cr^3+^ ions, while Pr^3+^ ions are used to enhance NIR emission under excitation at 222 nm as MgGa_2_O_4_: 0.005Cr, 0.003Pr. The concentration quenching was governed with the electric dipole–quadrupole interaction, while the afterglow mechanism was carefully revealed as the energy-splitting occurs from trivalent Cr^3+^ ions. We observed the emission at 709 nm decreased with increasing temperature, while the intensity of the satellite signal at 650 nm was increased first and then decreased, due to the intervalence charge transfer occurred among the Cr^3+^ ions. Ratiometric temperature sensing at 303–528 K was realized with the LIR at 709 and 650 nm under excitation at 222 nm with superb linearity, high absolute sensitivity and accuracy. Thus, the optical phenomenon is used with the ratiometric temperature sensing from the energy-splitting emission of trivalent Cr ions.

## Data availability

Data are available on request from the corresponding author.

## Author contributions

Tian-Qi Zhao: writing – original draft, writing – review & editing, conceptualization, data curation, formal analysis, validation. Renagul Abdurahman: funding acquisition, writing – review & editing, conceptualization, investigation, supervision. Xue-Bo Yin: writing – original draft, writing review & editing, funding acquisition, conceptualization, investigation, supervision.

## Conflicts of interest

There are no conflicts to declare.

## Supplementary Material

RA-014-D4RA04618H-s001
