# Supplementary material for: Energy-splitting from persistent luminescence nanoparticles with trivalent Cr ions for ratiometric temperature sensing
Source: RSC Adv. 2024 Aug 29;14(38):27514–9. doi: 10.1039/d4ra04618h (PMC11360775; doi:10.1039/d4ra04618h)
Supplement: RA-014-D4RA04618H-s001 [file RA-014-D4RA04618H-s001.pdf]

Supplementary Information (SI) for RSC Advances.
This journal is © The Royal Society of Chemistry 2024

Electronic supplementary information

Energy-splitting from persistent luminescence nanoparticles with trivalent Cr ions for ratiometric temperature sensing

Tian-Qi Zhao [a], Renagul Abdurahman [a, *], Xue-Bo Yin [b, *]

a. Xinjiang Key Laboratory of Novel Functional Materials Chemistry; College of Chemistry and Environmental Science, Kashi University, Kashi Xinjiang 844000 China.

**Corresponding author: renagul111@aliyun.com*

b. Institute for Frontier Medical Technology, College of Chemistry and Chemical Engineering, Shanghai University of Engineering Science, Shanghai, 201620 China.

**Corresponding author: xbyin@nankai.edu.cn or xbyin@sues.edu.cn*

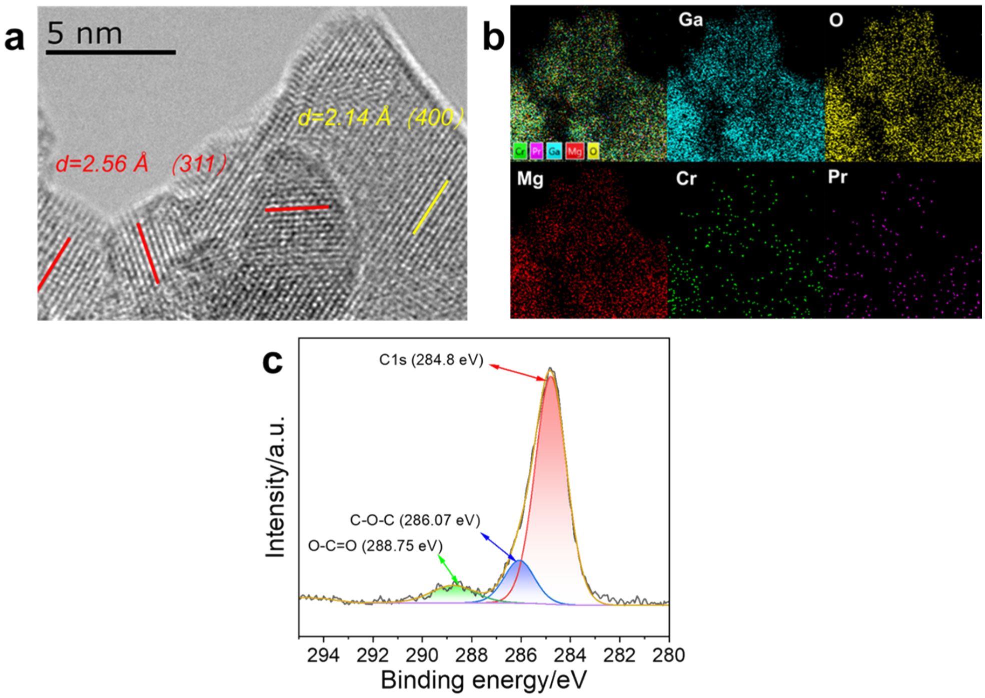

Fig. S1 (a) High-resolution TEM image and (b) elemental mapping of MGCP. (c) High-resolution XPS spectrum of carbon pollution in MGCP.

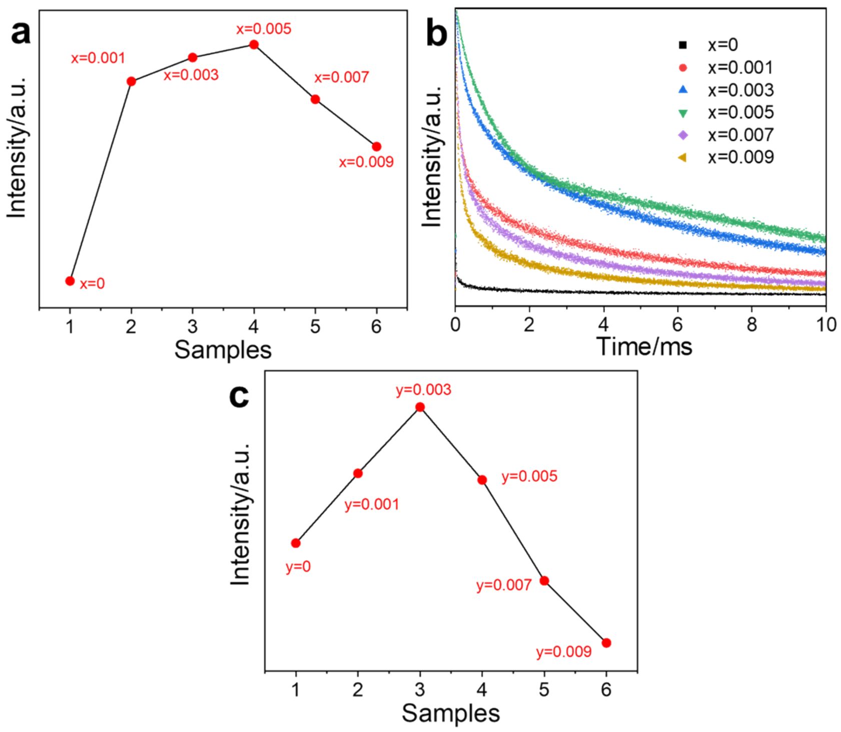

Fig. S2 (a) The emission intensity and (b) afterglow lifetime within 10 ms patterns for GO: x Cr^{3+} at 709 nm under excitation at 222 nm. (c) The emission intensity for MGO: 0.005 Cr^{3+}, yPr^{3+} at 709 nm under the excitation at 222 nm.

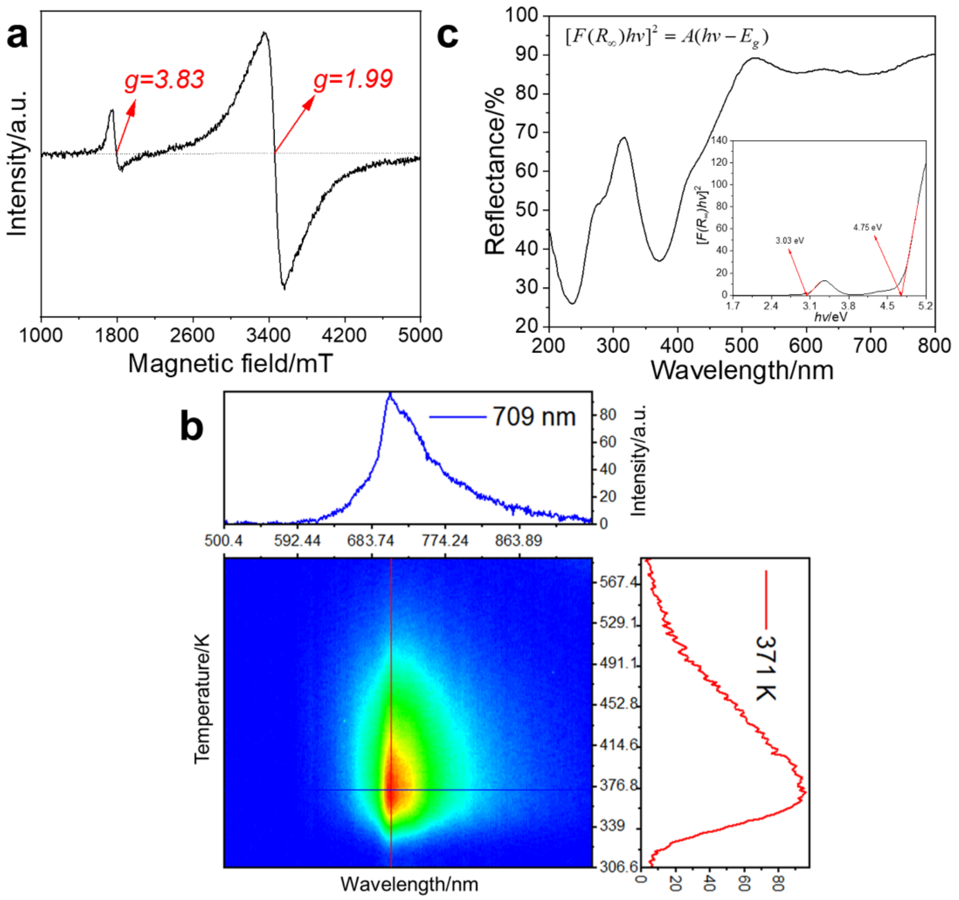

Fig. S3 (a) The EPR spectrogram, (b) thermoluminescence pattern, and (c) DRS spectrum (inset is relationship between $[F(R_\infty)hv]^2$ and hv) of MGCP.

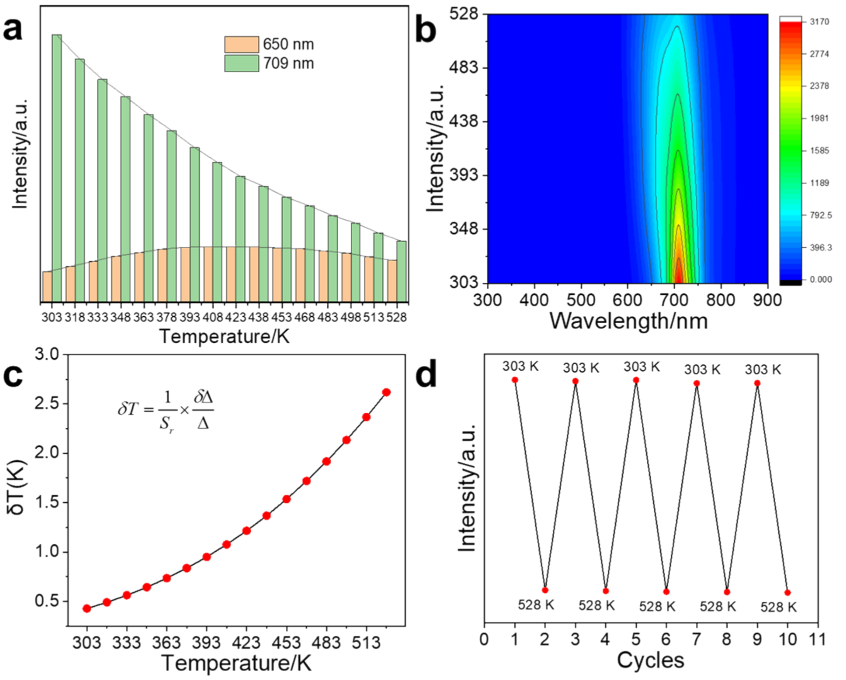

Fig. S4 (a) Histogram of emission intensity at 650 and 709 nm for MGCP at 222 nm excitation. (b) The emission contour graph, (c) accuracy, and (d) LIR values with the cycles of MGCP under 222 nm excitation at the temperature from 303 to 528 K.

Table S1 Comparison of LIR-based materials for temperature sensing

Host	Emission center	Temperature/ K	S_a (%K^{-1})	S_r (%K^{-1})	Ref.
$Na_5Y_9F_{32}$	Ce^{3+}, Tb^{3+}	298-473	0.157	1.18 (473K)	1
g-C_3N_4/$BaWO_4$	Yb^{3+}, Er^{3+}	293-393	1.07 (293K)	1.47 (393K)	2
Na_2MoO_4	Er^{3+}, Yb^{3+}	303-563	1.38 (563K)	1.21 (303K)	3
$NaY(WO_4)_2$	Er^{3+}, Yb^{3+}	293-503	0.9 (503K)	1.2 (293K)	4
$MgGa_2O_4$	Mn^{2+}, Mn^{4+}	80-500	8.15 (500K)	1.64 (260K)	5
γ-Ga_2O_3	Cr^{3+}, Ba^{2+}	300-420	3.40(-)	1.50 (300K)	6
$MgGa_2O_4$	Cr^{3+}, Zn^{2+} (MS1)	298-323	2.02(298K)	3.05(323K)	7
$MgGa_2O_4$	Cr^{3+}, Pr^{3+}	303-528	4.18 (303K)	1.17 (303K)	This work

References

1. F. Xu, B. Zheng, H. Xia, J. Wang, H. Song and B. Chen, *J. Alloys Compd.*, 2021, **873**, 159790.
2. L. Xu, J. Liu, L. Pei, Y. Xu and Z. Xia, *J. Mater. Chem. C*, 2019, **7**, 6112-6119.
3. H. Chen, Y. Jin, F. Fang, H. Lin, Y. Li, G. Feng, Y. Xiong, F. Meng, L. Cao and H. Ren, *J. Lumin.*, 2024, **265**, 120244.
4. M. Lin, L. Xie, Z. Wang, B. S. Richards, G. Gao and J. Zhong, *J. Mater. Chem. C*, 2019, **7**, 2971-2977.
5. B. Zhu, L. Wang, Q. Shi, H. Guo, J. Qiao, C. e. Cui and P. Huang, *J. Alloys Compd.*, 2023, **948**, 169717.
6. T. Zhao, R. Abdurahman, Q. Yang, R. Aiwaili and X.-B. Yin, *J. Mater. Chem. C*, 2024, **12**, 498-507.
7. T. Zhao, W. Deng, R. Abdurahman, S. Wu, X.-B. Yin, M. Tursun and J. Gao, *Inorg. Chem. Commun.*, 2024, **168**, 112908.
